# Macrophage NPM1 drives anti-tumor immunity via the STAT3-CCRL2 axis

**DOI:** 10.3389/fimmu.2026.1748791

**Published:** 2026-07-22

**Authors:** Lei Hong, Yue Yang, Yuwen Han, Nan Ouyang, Minxuan Sun, Rongchuan Zhao

**Affiliations:** 1 School of Biomedical Engineering (Suzhou), Division of Life Sciences and Medicine, University of Science and Technology of China, Hefei, China; 2 Suzhou Institute of Biomedical Engineering and Technology, Chinese Academy of Science, Suzhou, China; 3 Department of Chemistry, College of Sciences, Shanghai University, Shanghai, China

**Keywords:** CCRL2, NPM1, stat3, T cell, TAMs polarization, tumor immunity

## Abstract

**Background:**

Although nucleophosmin (NPM1) is a well-characterized oncogene in acute myeloid leukemia, much less is known about its role in solid tumor immunity. Here, we examined the function of NPM1 in regulating anti-tumor immunity within the tumor microenvironment.

**Methods:**

The proportion and functional changes of macrophages and T cells were detected by flow cytometry, the downstream regulatory genes of *NPM1* were detected by RNA-seq, the binding ability of NPM1 and STAT3 was analyzed by Chip and luciferase assay.

**Results:**

NPM1 inhibition or genetic deficiency promoted tumor growth in MC38 colon cancer and LLC lung cancer mice models. *Npm1^+/-^* mice exhibited enhanced infiltration and polarization of immunosuppressive M2-like tumor-associated macrophages (TAMs) and impaired CD8^+^ T cell function. Mechanistically, NPM1 bound to p-TAT3/STAT3 and promoted transcription of CCRL2, a key regulator of macrophage polarization. Overexpression of *Npm1* in mice suppressed tumor growth, reduced M2 TAMs polarization, and enhanced CD8^+^ T cell-mediated anti-tumor immunity.

**Conclusion:**

Our findings revealed that NPM1 protects against tumor progression by regulating TAMs polarization and T cell function via the STAT3-CCRL2 axis.

## Introduction

The tumor microenvironment (TME) refers to the non-cancer cells and components present in a tumor, as well as the proteins they produce and release (1, 2). The continuous interaction between tumor cells and the tumor microenvironment plays a decisive role in the occurrence, progression, metastasis of tumors and their response to treatment (3–5). Tumor-associated macrophages (TAMs) are the immune cells with the largest proportion in the TME (6). It plays an important regulatory role in the progression of cancer (7–9). The increased abundance of TAMs usually leads to an immunosuppressive tumor micro-environment and a poorer patient prognosis (10, 11). During tumor progression, under the stimulation of various factors in the complex TME, TAMs gradually transitions from being dominated by M1-type TAMs with pro-inflammatory phenotypes to M2-type TAMs with immunosuppressive phenotypes (12). M2-type TAMs are conducive to malignant angiogenesis, tumor cell proliferation and metastasis (13). Given the pro-tumorigenic role of M2-type TAMs, targeting their polarization is a rational approach for cancer immunotherapy.

Nucleophosmin (NPM1) is a multifunctional nucleoprotein that plays a key role in the regulation of cellular homeostasis and the occurrence of diseases (14–16). Mutations in the NPM1 gene are associated with various hematological malignancies, including acute myeloid leukemia (AML) (17–19). Furthermore, NPM1 is highly expressed in many solid tumors, including colorectal, gastric, hepatocellular and prostate malignancies (14, 20). And NPM1 has been demonstrated to exert a tumor-promoting effect by inhibiting apoptosis and promoting cell proliferation (20–22). In addition to directly promoting tumor cell growth, NPM1 could up-regulate the transcription of PD-L1 in triple-negative breast cancer (TNBC) cells, thereby inhibiting T cell activity in TNBC (23). NPM1 in tumor cells could also promote tumor immune evasion by inhibiting IRF1-mediated antigen presentation, thereby damaging tumor immunogenicity and reprogramming the immunosuppressive TME (14). Emerging studies establish NPM1 as a critical regulator of immune cells, demonstrating that it alleviates inflammatory bowel disease by regulating mitochondrial metabolism via the p65-TFAM axis in ILC3s (19) and enhances glycolytic metabolism through its interaction with KDM5b in macrophages during myocardial infarction (24). Nevertheless, its function within TAMs remains unexplored. Therefore, the potential contribution of NPM1 in TAMs to tumor progression and immune evasion deserves further study.

In this study, we found that NPM1 inhibition or genetic deficiency promotes tumor progression by enhancing M2-type polarization of TAMs and inhibiting anti-tumor immunity of CD8^+^ T cells. Mechanistically, we found that NPM1 enhances the transcription of CCRL2 by binding to transcription factor p-STAT3 and STAT3. More conclusive evidence indicates that overexpression of NPM1 could inhibit tumor progression by suppressing M2 polarization of TAMs and enhancing anti-tumor immunity of CD8^+^ T cells. Therefore, our study indicates that NPM1 enhances the anti-tumor function of TAMs and CD8^+^ T cells through the STAT3-CCRL2 axis.

## Methods

### 1 Animal models

All animal studies were reviewed and approved by the Animal Ethics committee, Suzhou Institute of Biomedical Engineering and technology Chinese Academy of Sciences (2024-C005). Generation of *Npm1^+/−^* and *Npm1^UTR+/−^* mice were previously described (19). Animals were housed under a 12-hour light/12-hour dark cycle with free access to food and water.

### 2 Cell culture and lentiviral infection

The mouse colon adenocarcinoma cell line MC38, mouse lung carcinoma cell line lewis lung carcinoma (LLC) and human monocytes THP-1 were obtained from the Cell Bank of the Chinese Academy of Sciences (Shanghai, China). MC38 and LLC were maintained in DMEM medium (Biosharp, Hefei, China) with 10% fetal bovine serum (FBS, Vazyme, Nanjing, China) and 1% penicillin–streptomycin mixture (Biosharp, Hefei, China). THP-1 were maintained in RPM1–1640 medium (Biosharp, Hefei, China) with 10% fetal bovine serum and 1% penicillin–streptomycin mixture. All the cells were incubated at 37 °C with 5% CO_2_.

To generate *NPM1* knockdown THP-1, MC38 and LLC cells, cells were stably transfected with a lentiviral vector encoding GFP-Puro- *NPM1* short hairpin RNA (shRNA, GenePharma, Shanghai). An empty vector virus served as a control. Stable cell lines were obtained through 2 μg/mL puromycin screening.

### 3 Tumor models and treatments

MC38 (1 × 10^6^) or LLC (5 × 10^5^) were implanted subcutaneously in the left groin of 6-8-week-old male and female *Npm1^+/−^* and *Npm1^UTR+/−^* mice. Tumor volumes were monitored using digital calipers every 2 days, and the volume was calculated using the following formula: V = (*L* × *W*²)/2, *L* represents the longest diameter and *W* represents the shortest diameter perpendicular to the length. Two weeks after injection, mice were euthanized via intraperitoneal injection of pentobarbital sodium at a dose of 200 mg/kg for subsequent histological and molecular analysis. For NPM1 inhibitor treatment, tumor cells were implanted (25) taneously in the left groin of 6-8-week-old male and female C57 BL/6J mice. 5 days later, 25μg NSC348884 (MCE, Shanghai, China) was administered via intraperitoneal injection one day before the inoculation of tumor cells. For the macrophage depletion experiment, mice received a tail vein injection of 100 μL clodronate liposomes (CL, Yeasen, Shanghai, China) one day prior to tumor inoculation, with subsequent booster injections administered every 7 days.

### 4 Flow cytometry

Single-cell suspensions were prepared from tumor tissues as described previously (25). The cells were stained using viability dye staining (Cat# 65-0865-18, eBioscience, USA). Then the cells were blocked with CD16/32 (Cat# 101301, BioLegend, USA) for 10 min at room temperature, followed by another 30 mins incubation with conjugated antibodies for extracellular markers at 4 °C. For intracellular cytokine detection, cells were stimulated *in vitro* with PMA, ionomycin and BFA for 4 h at 37 °C before FACS analysis. After stimulation, the cells were stained for surface markers and cytokines with Intracellular Fixation and Permeabilization Buffer Set (Cat# 88-8824-00, eBioscience, USA). Antibodies are used as follows: anti-CD11b FITC (Cat# 101205, BioLegend, USA), anti-CD45 BV785 (Cat# 157223, BioLegend, USA), anti-F4/80 PECY7 (Cat# 123113, BioLegend, USA), anti-CD86 PE (Cat# 159204, BioLegend, USA), anti-CD206 APC (Cat# 141707, BioLegend, USA), anti-CD3 FITC (Cat# 100204, BioLegend, USA), anti-CD4 APC (Cat# 100412, BioLegend, USA), anti-CD8 PE (Cat# 162304, BioLegend, USA), anti-CD8 PECY7 (Cat# 162312, BioLegend, USA), anti-IFNγ PE (Cat# 505807, BioLegend, USA), anti-TNFα FITC (Cat# 506304, BioLegend, USA), anti-Granzyme B APC (Cat# 372204, BioLegend, USA), macrophages (CD45^+^ CD11b^+^ F4/80^+^) were sorted using a FACS Aria Fusion flow cytometer (BD Biosciences).

### 5 Stimulated macrophages with tumor-conditioned medium

LLC cells were cultured in 10 cm dishes. When the cell density reached 80–90%, the medium was replaced with fresh medium containing 2% FBS, and the cells were further cultured for 12 hours. The collected medium was centrifuged at 3000 rpm for 10 minutes, and the supernatant is filtered through a 0.45 μm filter and then frozen at -80 °C for subsequent experiments. Bone marrow-derived macrophages (BMDMs) were isolated from the *Npm1^+/-^* and *Npm1^UTR+/−^* mice, cells were collected and filtered through 70-μm strainers, the red blood cells were removed by RBC lysis. Then the cells were differentiated for 7 days in RPMI-1640 with 10% FBS and 50 ng/mL M-CSF. THP-1 cells were treated with 100ng/mL PMA (Sigma, USA) for 24 h. Then BMDMs and THP-1 were treated with LPS (Sigma, USA), IL-4 (PeproTech, USA) tumor-conditioned medium for 24 h, RNAs were collected for detecting the markers of macrophage polarization.

### 6 Cell viability assay

Proliferation assays were conducted using *Npm1*-knockdown and control MC38 and LLC cells. Cells were seeded into 96-well plates and incubated for 3 days. For BMDMs, cells were treated with MC38 tumor conditioned medium for 3 days. Cell viability was subsequently determined using the CCK-8 assay.

### 7 qPCR

Total RNA was isolated from cells using FreeZol Reagent kit (Vazyme, Nanjing, China) according to the manufacturer’s instructions. The concentration and purity of RNA were detected using a Nanodrop 2000 spectrophotometer (Thermo Fisher Scientific, USA). Subsequently, total RNA was reverse transcribed into cDNA using HiScript II Q Select RT SuperMix with gDNA Eraser (Vazyme, Nanjing, China), which effectively eliminated genomic DNA contamination. The relative mRNA expression levels of target genes were analyzed by quantitative real-time PCR (qPCR) using SYBR Green PCR master mix (Vazyme, Nanjing, China). Sequences of primers for q-PCR were listed in **Supplementary Table 1**.

### 8 RNA sequencing

CD45^+^ CD11b^+^ F4/80^+^ macrophages were sorted from tumors of Npm1^+^/^+^ and Npm1^+^/^-^ mice. Total RNA was isolated using the RNeasy Mini Kit (Qiagen) with DNase I digestion. RNA quality was verified by NanoDrop and Agilent 2100 Bioanalyzer, samples with RIN values between 8.2 and 9.8 were selected for RNA-seq library construction and sequencing. cDNA was synthesized with the SMARTer^®^ Ultra™ Low Input RNA Kit v4 (Takara). cDNA libraries were prepared via fragmentation, blunt-end repair, A-tailing, adapter ligation, and PCR amplification using AMPure XP beads for size selection. Paired−end sequencing was performed on an llumina NovaSeq 6000 platform.

### 9 Immunoprecipitation assay

1×10^7^ THP-1 cells were collected and washed twice with pre-cooled PBS, and 1 mL of IP lysis buffer (Beyotime, Cat # P0017) containing 1% PMSF and 1% protease inhibitor cocktail was added to each dish. The cells were lysed on ice for 30 minutes, and the lysate was centrifuged at 12,000 rpm for 15 minutes at 4 °C to collect the supernatant. The supernatant was incubated with anti-NPM1 antibodies (2 μg, Cat #A17983, ABclonal) or IgG for immunoprecipitation. Protein A/G agarose (MCE, China) was added to the lysate-antibody mixture and incubated with gentle agitation at 4°C for 3 h. Then immunocomplexes were subsequently washed with lysis buffer, followed by 40µL of 1 × SDS loading buffer. Western blot was used for detecting interacting proteins.

### 10 Western blot

After cell lysis, protein quantification was performed using the BCA assay. Following the addition of an appropriate volume of loading buffer and heat-induced denaturation at 95 °C, proteins were separated by SDS-PAGE according to their molecular weight and subsequently transferred onto a PVDF membrane. The membrane was then blocked with 5% non-fat milk and incubated with the corresponding primary antibody CCRL2 (1:1000, Cat# 66611-1-Ig, Proteintech, China), α-tubulin (1:5000, Cat# AY0796, Abways, China), STAT3 (1:1000, Cat# 12640S, CST, USA), p-STAT3 (1:1000, Cat# 9145S, CST, USA) and NPM1 (1:1000, Cat# A17983, Abclonal, China) overnight at 4 °C. After three washes with TBST, the appropriate secondary antibody was applied, and the membrane was incubated at room temperature for 1 hour. Finally, ECL reagent was added for chemiluminescent imaging.

### 11 ChIP assay

The ChIP assay was conducted using the ChIP-IT Kit (Beyotime, China). In brief, the cells were initially fixed with formaldehyde and subsequently lysed. To precipitate the DNA fragment, either 2 μg of anti-STAT3 or normal IgG were used. The DNA-protein complexes were then pulled down with magnetic beads and subjected to decross-linking. The extracted DNA samples were finally amplified using specific CCRL2 promoter primers for the sequences containing the binding site 5′-GCGTG-3′.

### 12 Luciferase reporter assay

BMDMs that overexpressed STAT3 were transfected with the specified pGL3-luciferase reporter plasmid (GenePharma, Shanghai) that contained the CCRL2 promoter, along with the Renilla pRL-TK plasmid as the internal control. After incubation for 24–48 h, the cell lysates were subjected to luciferase activity analysis using the Dual-Luciferase Reporter Assay kit (Promega, USA), each sample has six replicates.

### 13 Statistical analysis

All experiments were carried out independently with at least three replicates. Statistical analyses were performed using GraphPad Prism 8.0 (GraphPad, San Diego, CA, USA). Data were presented as mean ± SEM. Significant differences between two groups were assessed by a non-paired t test or one-way ANOVA analysis. For comparisons between three or more groups, two-way ANOVA analyses were used. All statistical tests were two-tailed and a p-value of <0.05 was considered statistically significant.

## Results

### 1 NPM1 inhibitors promote tumor progression by inducing an immunosuppressive phenotype in macrophages

**Figure 1:**
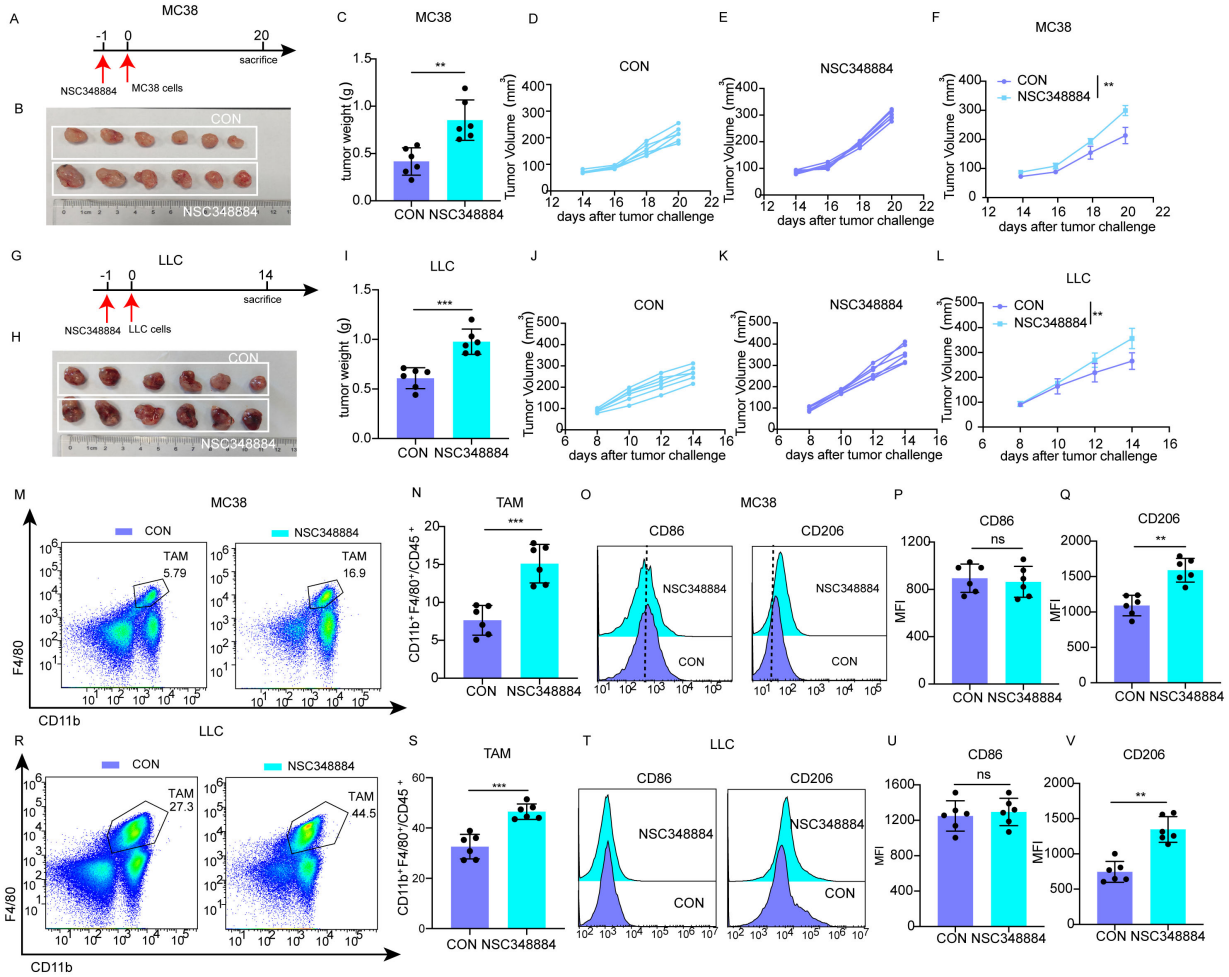
NPM1 inhibitors promote tumor progression by inducing an immunosuppressive phenotype in macrophages. **(A)** Schematic diagram of the subcutaneous tumor model (*n* = 6 per group). **(B)** Representative photographs of MC38 tumors from CON and NSC348884 treated mice (*n*= 6 per group). **(C)** Comparison of MC38 tumor weight from two groups at the end of the experiment. **(D–F)**. The growth curve of MC38 in CON and NSC348884 treated mice. **(G)** Schematic diagram of the LLC tumor model. **(H)** Representative image of tumor growth in CON and NSC348884 treated mice subcutaneously inoculated with LLC cells (*n*= 6 per group). **(I)** Comparison of LLC tumor weight from two groups at the end of the experiment. **(J–L)**. The growth curve of LLC in CON and NSC348884 treated mice. **(M, N)** Flow cytometry analysis of the percentage of TAMs in MC38 tumors, representative FACS plots **(M)** with quantification **(N)** were shown. **(O–Q)** Flow cytometry analysis of the MFI of CD86 and CD206 in TAMs, representative FACS plots **(O)**, quantification of CD86 **(P)** and CD206 **(Q)** were shown. **(R, S)** Flow cytometry analysis of the percentage of TAMs in LLC tumors, representative FACS plots **(R)** and quantification **(S)** were shown. **(T–V)** Flow cytometry analysis of the MFI of CD86 and CD206 in TAMs, representative FACS plots **(T)**, quantification of CD86 **(U)** and CD206 **(V)** were shown. Statistical differences were analyzed by unpaired t-test. **p < 0.01, ***p < 0.001.

To investigate the impact of inhibiting NPM1 in mice on tumor progression, a NPM1 inhibitor, NSC348884 was intraperitoneally injected into mice one day before inoculating tumor cells. To characterize the effect of inhibiting NPM1 across different tumor environments, we employed two distinct syngeneic tumor models: MC38 colon cancer and LLC lung cancer (**Figures 1A–L**). Consistent with our hypothesis, pharmacological inhibition of NPM1 significantly promoted tumor growth (**Figures 1B, H**), as evidenced by increased tumor size (**Figures 1C, I**) and weight (**Figures 1D–F, J–L**) in both models.

Given the pivotal role of TAMs in shaping the tumor immune landscape, we next characterized TAMs infiltration and polarization states (**Figures 1M–V**; **Supplementary Figure 1A**). Flow cytometric analysis revealed that NPM1 inhibition markedly enhanced TAMs infiltration (**Figures 1M, N, R, S**) and promoted an immunosuppressive phenotype, as indicated by increased expression of the M2-type marker CD206 (**Figures 1O–Q, T–V**). In contrast, the M1-type marker CD86 remained unaffected (**Figures 1P, U**).

**Figure 2:**
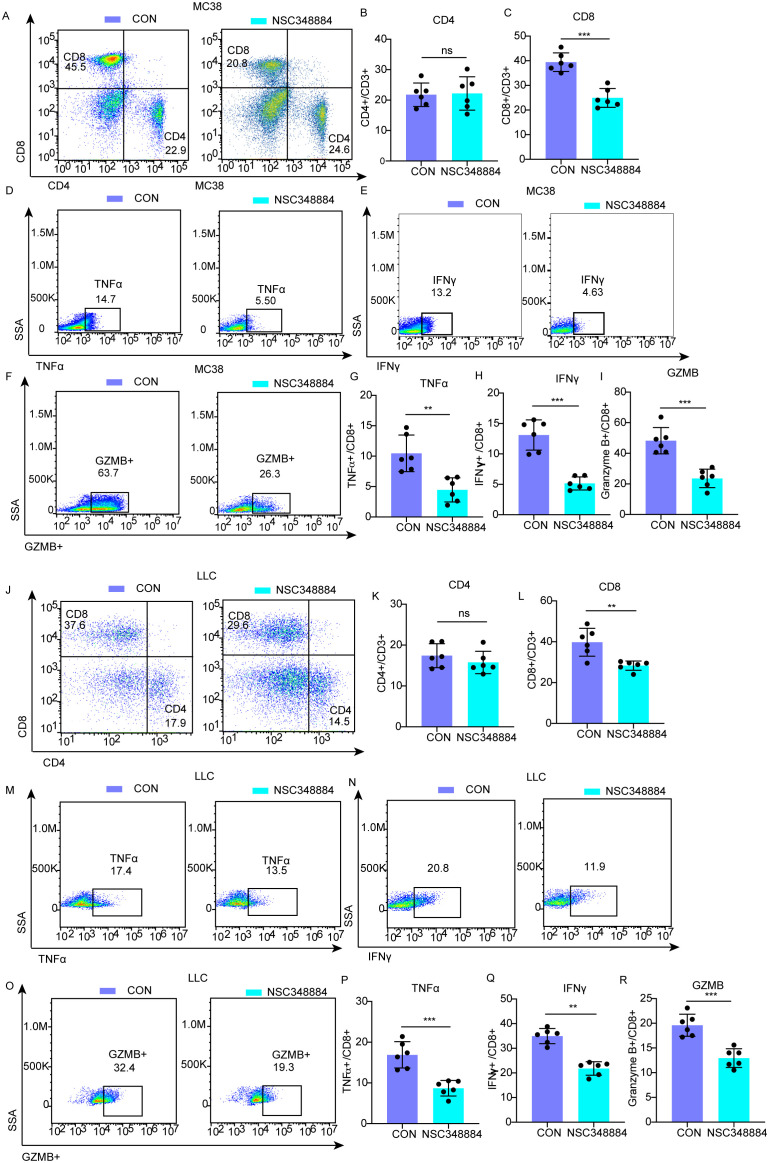
NPM1 inhibitor supresses the infiltration and function of CD8^+^ T cells. **(A–C)** Flow cytometric analysis of CD4^+^ and CD8^+^ T cell proportions in MC38 tumors. Representative flow plots **(A)** and corresponding quantitative results **(B, C)** are shown (n = 6 per group). **(D–I)** Flow cytometry analysis of the GZMB^+^/IFNγ^+^/TNFα^+^ CD8^+^ T cells in MC38 tumors, representative FACS plots **(D–F)** and quantification **(G–I)** were shown (n=6 per group). **(J–L)** Flow cytometry analysis of the percentage of CD4^+^ T cells and CD8^+^ T cells in LLC tumors, representative FACS plots **(J)** with quantification **(K–L)** were shown. **(M–R)** Flow cytometry analysis of the GZMB^+^/IFNγ^+^/TNFα^+^ CD8^+^ T cells in LLC tumors, representative FACS plots **(M–O)** and quantification **(P–R)** were shown. Statistical differences were analyzed by unpaired t-test, **p < 0.01, ***p < 0.001.

Since macrophage polarization critically influences T cell-mediated anti-tumor immunity, we subsequently analyzed T cell populations and their functional states (**Figures 2A–R**; **Supplementary Figure 1B**). Results demonstrated a significant reduction in CD8^+^ T cell infiltration following NPM1 inhibition (**Figures 2C, L**), while CD4^+^ T cell proportions remained unchanged (**Figures 2B, K**). Moreover, functional assessment of CD8^+^ T cells revealed substantial impairment of their cytotoxic capacity, with decreased production of TNF-α, IFN-γ, and granzyme B (**Figures 2D–I, M–R**). These findings collectively indicated that NPM1 inhibition promotes tumor progression by driving TAMs toward an immunosuppressive phenotype and subsequently impairing CD8^+^ T cell-mediated anti-tumor immunity.

### 2 NPM1 deficiency impairs anti-tumor immunity *in vivo*

**Figure 3:**
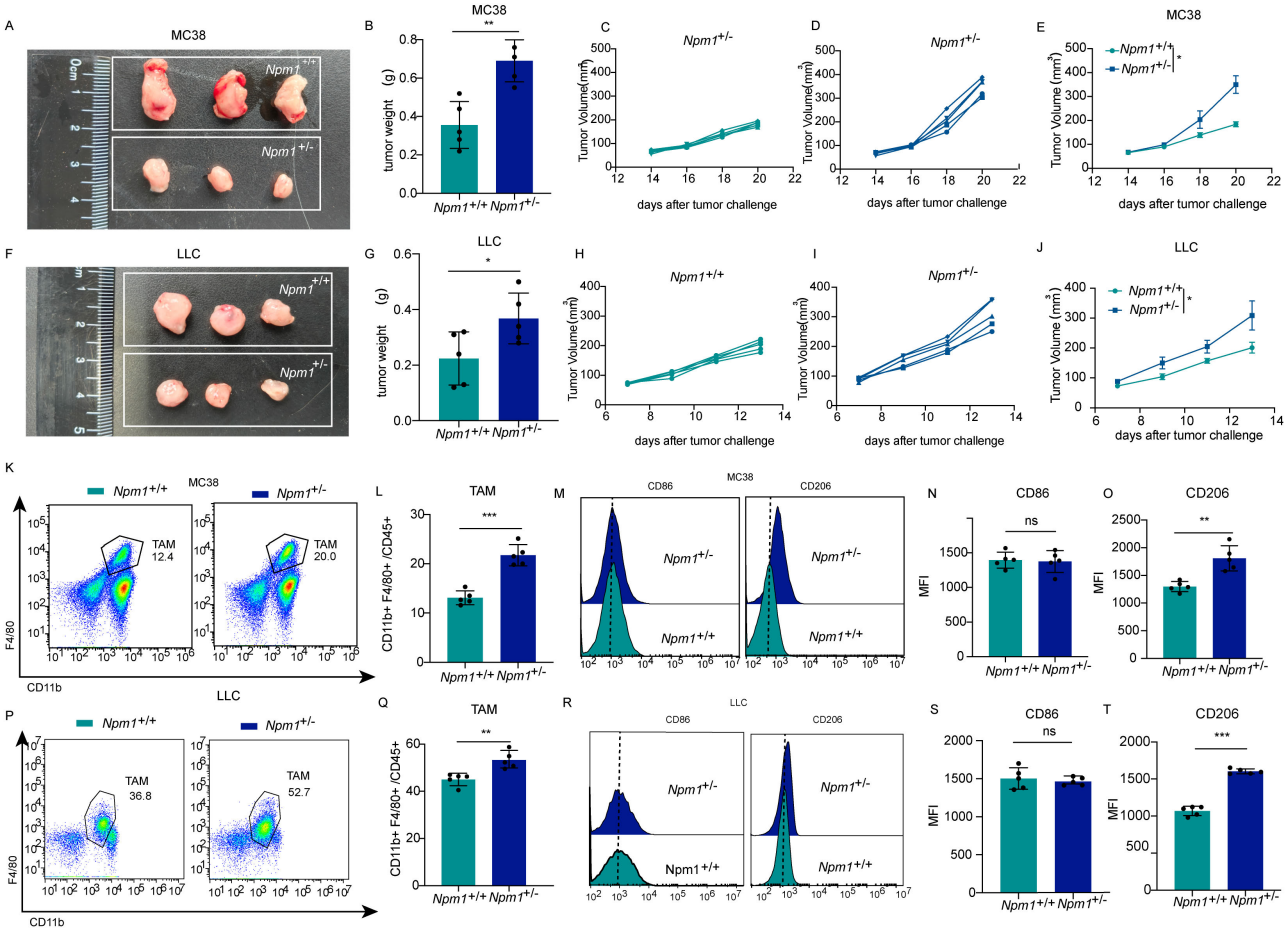
NPM1 deficiency impairs anti-tumor immunity *in vivo*. **(A)** Representative image of tumor growth in *Npm1*^+/+^ and *Npm1*^+/-^ treated mice subcutaneously inoculated with MC38 cells (*n*= 5 per group). **(B)** Comparison of MC38 tumor weight from two groups at the end of the experiment. **(C–E)**. The growth curve of MC38 cells in *Npm1*^+/+^ and *Npm1*^+/-^ treated mice. **(F)** Representative image of tumor growth in *Npm1*^+/+^ and *Npm1*^+/-^ treated mice subcutaneously inoculated with LLC cells (*n*= 5 per group). **(G)** Comparison of LLC tumor weight from two groups at the end of the experiment. **(H–J)**. The growth curve of LLC cells in *Npm1*^+/+^ and *Npm1*^+/-^ treated mice. **(K, L)** Flow cytometry analysis of the percentage of TAMs in MC38 tumors, representative FACS plots **(K)** with quantification **(L)** were shown. **(M–O)** Flow cytometry analysis of the MFI of CD86 and CD206 in TAMs, representative FACS plots **(M)**, quantification of CD86 **(N)** and CD206 **(O)** were shown. **(P, Q)** Flow cytometry analysis of the percentage of TAMs in LLC tumors, representative FACS plots **(P)** and quantification **(Q)** were shown. **(R–T)** Flow cytometry analysis of the MFI of CD86 and CD206 in TAMs of LLC tumor models, representative FACS plots **(R)**, quantification of CD86 **(S)** and CD206 **(T)** were shown. Statistical differences were analyzed by unpaired t-test. *p < 0.05, **p < 0.01.

Previous studies have shown that NPM1 expression in tumor cell is positively correlated with tumor progression (26, 27), and our *in vitro* results are consistent with this: Knockdown of NPM1 significantly inhibits tumor cell proliferation (**Supplementary Figures 2A–F**). However, this stands in contrast to our *in vivo* findings (**Figures 1A–L**), suggesting that systemic NPM1 inhibition may exert a greater influence on the tumor immune microenvironment than on tumor cell-intrinsic proliferation. To genetically validate the role of NPM1 in anti-tumor immunity, we utilized *Npm1*-haploinsufficient mice (*Npm1*^+/-^), as homozygous knockout is embryonically lethal. Consistent with pharmacological inhibition, genetic deficiency of *Npm1* significantly accelerated tumor growth in both LLC and MC38 models, as reflected by increased tumor size and weight (**Figures 3A–J**). Flow cytometric analysis of the TME revealed an increased TAMs proportion and a higher ratio of immunosuppressive CD206 positive TAMs in *Npm1*^+/-^ mice compared to wild-type controls (**Figures 3K–T**). Further analysis revealed that *Npm1* deficiency promoted BMDM proliferation, impaired CD8^+^ T cell infiltration and inhibited their cytotoxic function, evidenced by reduced TNF-α^+^, IFN-γ^+^, and granzyme B^+^ subsets, without affecting CD4^+^ T cells (**Supplementary Figures 3A–S**). To further verify whether NPM1 regulates tumor progression through macrophages, we performed macrophage depletion using CL in tumor-bearing mice (**Supplementary Figure 4A**). CL treatment significantly suppressed tumor growth and reduced the final tumor weight and volume in tumor-bearing mice. Most importantly, no significant difference in tumor size was observed between *Npm1^+/-^* mice and wild-type controls with CL treatment (**Supplementary Figures 4B–D**).

### 3 NPM1 promotes CCLR2 transcription by binding to STAT3.

**Figure 4:**
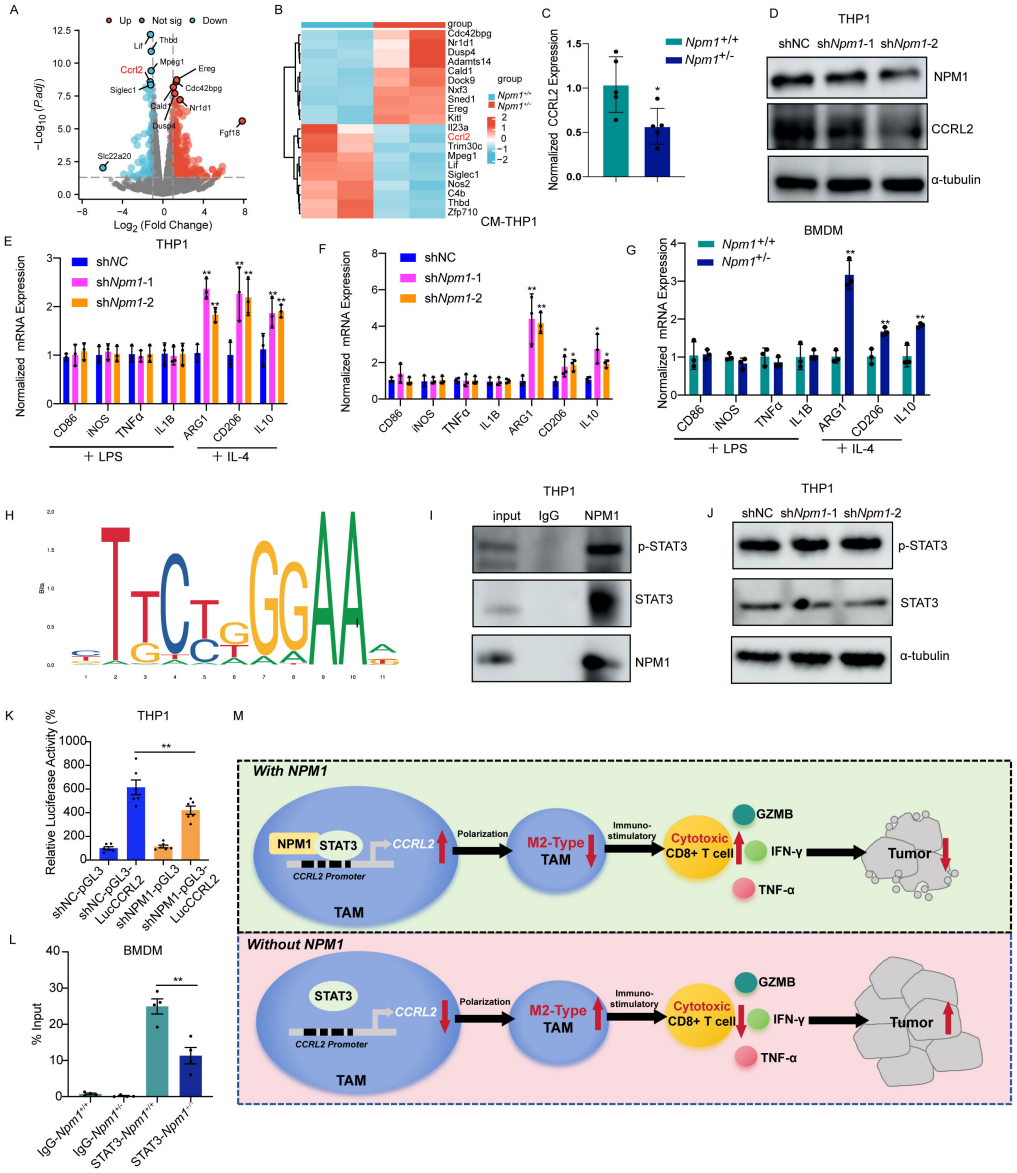
NPM1 promotes CCLR2 transcription by binding to STAT3. **(A)** Volcano map of DEGs in TAMs isolated from *Npm1^+/−^* mice compared to that in *Npm1^+/+^* mice **(B)** Heatmap of Top 10 down-regulated genes in TAMs isolated from *Npm1^+/−^* mice compared to that in *Npm1^+/+^* mice (n = 2 individual mice). **(C)** CCRL2 mRNA expression in TAMs of *Npm1^+/−^* and *Npm1^+/+^* mice. **(D)** Protein expression analysis of NPM1 and CCRL2 in sh*Npm1* and sh*NC* THP-1 cells. **(E)** qPCR assays were used to analyze the expression of M1-type or M2-type macrophage markers in THP-1cells treated with LPS or IL-4. **(F)** qPCR assays were used to analyze the expression of M1-type or M2-type macrophage markers in THP-1cells treated with tumor-conditioned medium. **(G)** qPCR assays were used to analyze the expression of M1-type or M2-type macrophage markers in BMDMs treated with LPS or IL-4. **(H)** Logo plot of the consensus binding motif of STAT3. **(I)** IP and IB of the interaction between NPM1 STAT3 and p-STAT3 in THP-1 cells. The samples were derived from the same experiment, and the blots were processed in parallel. **(J)** Protein expression analysis of p-STAT3 and STAT3 in sh*Npm1* and sh*NC* THP-1 cell. **(K)** CCRL2 reporter activity measured in THP-1 cells. **(L)** ChIP-qPCR assays of the binding efficiency of STAT3 to the CCRL2 promoter in BMDM cells. IgG served as the negative control. **(M)** The proposed model for the anti-tumor role of NPM1-STAT3-CCRL2 axis in TAMs. Statistical differences were analyzed by unpaired t-test, *p < 0.05, **p<0.01.

To elucidate the molecular mechanism underlying NPM1-mediated TAM function, we performed RNA sequencing on TAMs sorted from LLC tumors of *Npm1*^+/+^ and *Npm1*^+/-^ mice. Differential expression analysis identified CCRL2 as one of the most significantly down-regulated genes in *Npm1*-deficient TAMs (**Figures 4A, B**). C-C motif chemokine receptor-like 2 (CCRL2), a non-signaling atypical receptor first cloned from LPS-activated macrophages, has been revealed that could promote the immunostimulatory phenotype in macrophages, thereby licensing a potent antitumor T-cell response that leads to tumor rejection (28). This mechanism may account for the diminished M2 polarization of TAMs and the attenuated activation of antitumor CD8^+^ T cells observed in *Npm1*^+/-^ mice compared to *Npm1*^+/+^ mice. CCRL2 expression was markedly decreased in TAMs or BMDMs isolated from *Npm1*^+/-^ mice (**Figure 4C**; **Supplementary Figure 5A**). CCRL2 expression was substantially reduced upon *NPM1*-knockdown in THP-1 cells (**Figure 4D**). Given the established role of CCRL2 in regulating macrophage polarization, BMDMs and THP-1 cells were stimulated with LPS, IL-4 or tumor-conditioned medium to test macrophage polarization *in vitro*. The results showed that Npm1 knockdown promoted polarization toward M2-type macrophages, as the expression of M2 markers ARG1, CD206 and IL10 was significantly upregulated, whereas the expression of M1 markers CD86, iNOS, TNFα and IL1B remained unchanged (**Figures 4E–G**), suggesting that down-regulating CCRL2 may be a key factor of increased TAMs M2 polarization in *Npm1*^+/-^ mice.

As NPM1 frequently functions as a transcriptional cofactor, we sought to identify its partner transcription factor in the transcriptional activation of CCRL2. Promoter sequence analysis of CCRL2 nominated STAT3 as a putative regulator (**Figure 4H**). Furthermore, given the reported cooperation between NPM1 and STAT3 in transcriptional regulation in cancer cell (29), we sought to investigate this potential collaboration in the context of CCRL2 transactivation in macrophage. The interaction between NPM1 and STAT3, as well as p-STAT3 in macrophage was confirmed by co-immunoprecipitation (**Figure 4I**). While NPM1 knockdown did not alter the expression of STAT3 or p-STAT3 (**Figure 4J**). To further validate that the NPM1-STAT3 interaction promotes CCRL2 transcription, we conducted a promoter reporter assay in BMDMs from *Npm1*^+/+^ and *Npm1*^+/-^ mice. The results showed that STAT3 bound to CCRL2 promoter and enhanced CCRL2 transcription, while *Npm1* deficiency significantly attenuated the transcriptional efficiency of STAT3 on CCRL2 (**Figure 4K**). ChIP-qPCR experiments also demonstrated that *Npm1* deficiency significantly reduced STAT3 occupancy at the CCRL2 promoter (**Figure 4L**). Collectively, above results demonstrate that NPM1, in complex with STAT3, promotes CCRL2 transcription to orchestrate the M2 polarization of TAMs (**Figure 4M**).

### 4 Overexpression of *Npm1* in mice promotes antitumor immunity *in vivo*

**Figure 5:**
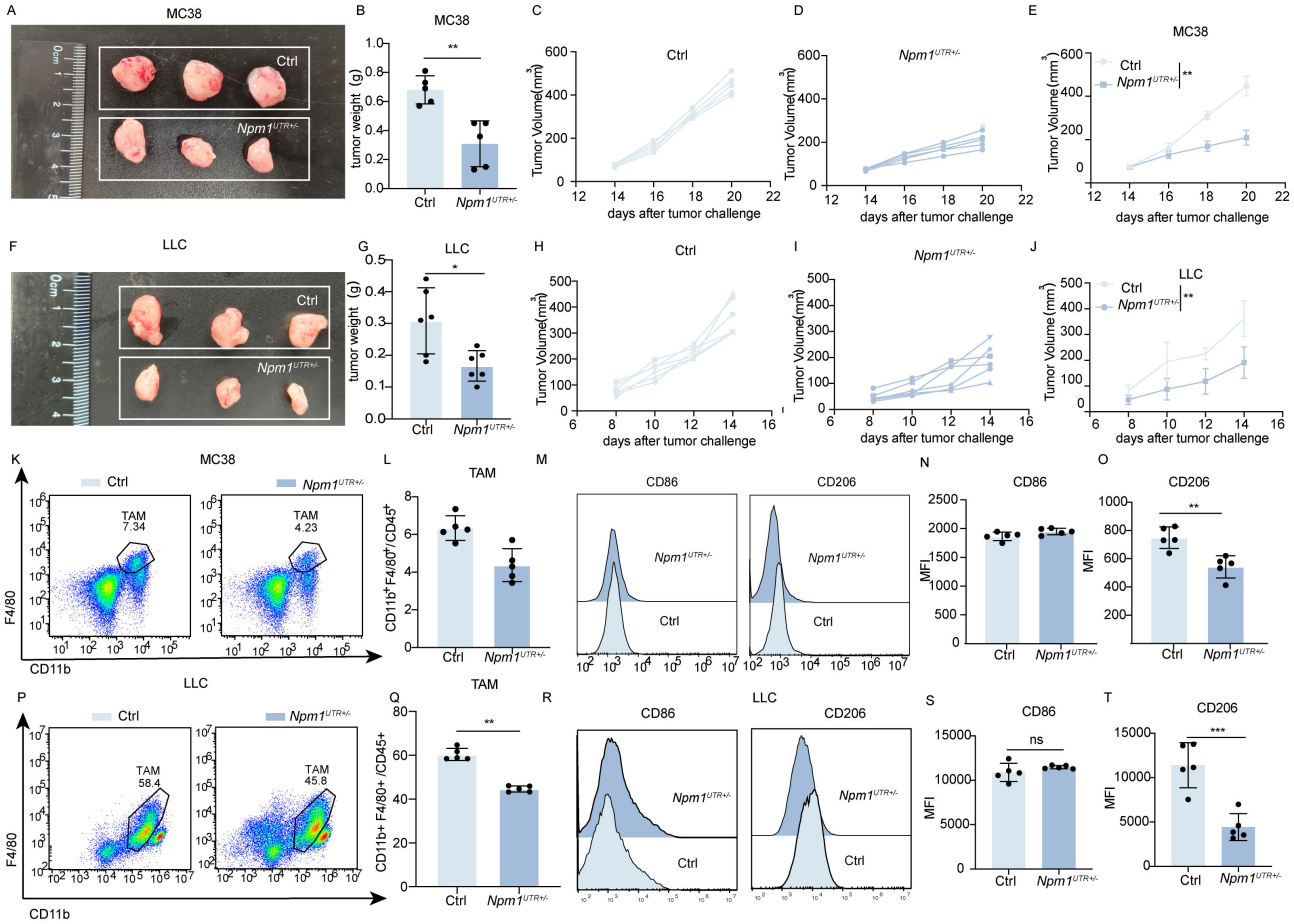
Overexpression of *Npm1* in mice promotes antitumor immunity *in vivo*. **(A)** Representative image of tumor growth in Ctrl and *Npm1^UTR-/-^* treated mice subcutaneously inoculated with MC38 cells (*n*= 6 per group). **(B)** Comparison of MC38 tumor weight from two groups at the end of the experiment. **(C–E)**. The growth curve of MC38 cells in Ctrl and *Npm1*^UTR-/-^treated mice. **(F)** Representative image of tumor growth in Ctrl and *Npm1*^UTR-/-^ treated mice subcutaneously inoculated with LLC cells (*n*= 6 per group). **(G)** Comparison of LLC tumor weight from two groups at the end of the experiment. **(H–J)**. The growth curve of LLC cells in Ctrl and *Npm1*^UTR-/-^ treated mice. **(K, L)** Flow cytometry analysis of the percentage of TAMs in MC38 tumors, representative FACS plots **(K)** with quantification **(L)** were shown. **(M–O)** Flow cytometry analysis of the MFI of CD86 and CD206 in TAMs, representative FACS plots **(M)**, quantification of CD86 **(N)** and CD206 **(O)** were shown. **(P, Q)** Flow cytometry analysis of the percentage of TAMs in LLC tumors, representative FACS plots **(P)** and quantification **(Q)** were shown. **(R–T)** Flow cytometry analysis of the MFI of CD86 and CD206 in TAMs of LLC tumor models, representative FACS plots **(R)**, quantification of CD86 **(S)** and CD206 **(T)** were shown. Statistical differences were analyzed by unpaired t-test. *p < 0.05, **p < 0.01, ***p < 0.001.

Above results indicating that *Npm1* deficiency is a critical driver of tumor progression, more experiments in *Npm1* overexpressed mice were performed to verified it. *Npm1*^UTR-/-^ mice with *Npm1* overexpression were generated, then MC38 and LLC were inoculated subcutaneously into control and *Npm1*^UTR-/-^ mice. *Npm1*^UTR-/-^ mice exhibited significantly reduced tumor formation in both MC38 and LLC tumors (**Figures 5A–J**). *Npm1*^UTR-/-^ mice also exhibited a substantial reduction in TAMs infiltration (**Figures 5K, L, P, Q**). Furthermore, the infiltrated TAMs displayed a decreased M2-polarized phenotype in *Npm1*^UTR-/-^ mice compared to control mice (**Figures 5M–O, R–T**). In contrast to *Npm1*^+/-^ mice, the infiltration of CD8^+^ T cells in *Npm1*^UTR-/-^ was increased (**Supplementary Figures 6A–C, J–L**), and the proportion of granzyme B^+^/IFNγ^+^/TNFα^+^ CD8^+^ T cells were also increased (**Supplementary Figures 6D–I, M–R**), indicated an enhanced tumor killing function of CD8^+^ T cells. In parallel, we validated the expression pattern of CCRL2 in BMDMs isolated from *Npm1^UTR-/-^* mice, and the data demonstrated a significant upregulation of CCRL2 in these cells (**Supplementary Figure 5B**). Moreover, after stimulation with tumor-conditioned medium, a notable reduction was found in the mRNA expression of M2 polarization markers in *Npm1^UTR-/-^* mouse-derived BMDMs, whereas the expression of M1 polarization markers was not significantly altered (**Supplementary Figure 5C**). Collectively, our results establish NPM1 as a key regulator of TAMs function and a crucial player in anti-tumor immunity.

## Discussion

In this study, NPM1 was demonstrated as a critical regulator of anti-tumor immunity by modulating TAMs polarization and CD8^+^ T cell function within the tumor microenvironment. The disruption of NPM1-STAT3-CCRL2 signaling axis in TAMs was demonstrated to promote an immunosuppressive TAMs phenotype and accelerates tumor progression. Conversely, genetic overexpression of *Npm1* reinforces anti-tumor immunity and suppresses tumor growth. These findings establish NPM1 as a pivotal node in the immune response against cancer and suggest its potential as a therapeutic target.

Previous studies showed that tumor-intrinsic *Npm1* knockdown suppressed proliferation, consistent with its known oncogenic functions in cellular growth and survival (30, 31). while systemic *Npm1* deficiency unexpectedly promoted tumor progression. NPM1 mediated activation of the CD274 promoter and expression of programmed death ligand-1 (PD-L1) promotes lung cancer progression (26). *Npm1* deficiency in ILC3 promotes progression of colorectal cancer by regulating mitochondrial oxidative phosphorylation (19). This apparent paradox underscores the critical balance between NPM1’s tumor-cell-intrinsic functions and its immune-regulatory roles within the TME. The contrasting effects observed in tumor cells versus immune cells highlight the importance of cell-type-specific targeting strategies for future therapeutic development.

A key finding of our study is the identification of the NPM1-STAT3-CCRL2 axis as a fundamental mechanism regulating macrophage polarization. STAT3 is a well-established transcriptional regulator in cancer immunology, integrating signals from various cytokines and growth factors to modulate immune cell function (32, 33). Previous study showed that p-STAT3 physically binds to NPM1 and that this interaction facilitates the nuclear translocation of p-STAT3, while NPM1 in turn helps retain p-STAT3 in the nucleus to sustain its transcriptional activity (29). Our co-immunoprecipitation results also validated the physical interaction between NPM1 and STAT3/p-STAT3 protein in TAM, which means that NPM1 likely promotes the transcription of STAT3 target genes by enhancing the nuclear accumulation of p-STAT3 and facilitating the assembly of the transcriptional machinery at target gene promoters. In this study, NPM1 was confirmed to interact with STAT3 and promotes its transcriptional activity toward the CCRL2 promoter, providing a mechanistic link between nucleolar function and immune gene regulation. Our study revealed that CCRL2 had a potentiating anti-tumor T-cell responses, which was consist with previous study in murine melanoma (28). CCRL2 deletion in macrophages impairs the polarization of M1 macrophages induced by LPS/IFN-γ (34, 35). CCRL2 has emerged as an important chemokine receptor-like protein involved in shaping macrophage phenotypes in inflammatory diseases (34, 36). Our work extends its significance to tumor immunology, identifying it as a downstream effector of NPM1 signaling in TAMs polarization. *Npm1* deficiency and NSC348884 also led to an impairment of CD8^+^ T cell infiltration and its cytotoxic function, creating an immune-permissive environment for tumor growth. Notably, given the potential off-target effects inherent to the small-molecule NPM1 inhibitor NSC348884, rigorous genetic manipulation approaches such as RNAi-mediated silencing or CRISPR/Cas9-mediated gene editing are required to further validate the specific regulatory role of NPM1 in the activation, polarization and functional modulation of immune cells within the tumor microenvironment. CCRL2 deletion in macrophages also reduces the *in vitro* activation of antigen-specific OT1 CD8^+^ T cells and inhibition of anti-tumor T cell responses (28). Our findings are consistent with the growing evidence that the polarization state of macrophages directly affects the recruitment, activation and effector function of T cells within tumors.

In this study, we provide compelling evidence that enhancing NPM1 function represents a viable strategy to potentiate anti-tumor immunity. The significant tumor suppression observed in *Npm1*^UTR-/-^ mice, coupled with the favorable remodeling of the immune microenvironment, suggests that therapeutic strategies aimed at augmenting NPM1 activity in immune cells could complement existing immunotherapies. Particularly promising is the demonstration that NPM1 overexpression simultaneously reduces immunosuppressive TAMs populations and enhances CD8^+^ T cell function—two key hallmarks of effective anti-tumor immunity.

Another notable limitation should also be addressed that although we verified partial candidate genes via qPCR, this limited validation cannot fully compensate for insufficient biological replicates in RNA-seq analysis, which may restrict the reliability of transcriptomic profiling results.

In conclusion, our study identifies NPM1 as a central regulator of tumor immunity, which orchestrates TAM polarization and CD8+ T cell function through the STAT3-CCRL2 axis. These findings significantly expand our understanding of nucleolar proteins in immune regulation and provide a compelling rationale for exploring NPM1-centered immunotherapeutic strategies. A limitation of this work is the absence of direct CCRL2 rescue or loss-of-function experiments; while our findings identify CCRL2 as a key downstream effector of the NPM1-STAT3 axis, definitive functional validation of CCRL2 dependency in NPM1-mediated tumor immune regulation is warranted in future studies. Future investigations into the tissue-specific activation of NPM1 may unlock novel combination therapeutic strategies to overcome resistance to current cancer immunotherapies, thus providing new avenues for clinical translation.

## Data Availability

The data presented in the study are deposited in the GEO repository, accession number GSE339161.

## Supplementary material

Supplementary Figure 1 Flow cytometry gating strategy. **(A)** Gating strategy for macrophages. **(B)** Gating strategy for T cells.

Supplementary Figure 2 *Npm1* knockdown suppresses tumor cell proliferation *in vitro*. **(A, C)** Western blot analysis of NPM1 protein levels in sh*NC* and sh*Npm1* MC38 **(A)** and LLC **(C)** cells. **(B, D)** Quantitative statistical analysis of NPM1 protein expression. **(E, F)** Cell viability of MC38 and LLC cells with *Npm1* knockdown versus control. Statistical differences were analyzed by unpaired t-test. **p < 0.01, ***p < 0.001.

Supplementary Figure 3 NPM1 deficiency inhibite the infiltration and function of CD8^+^ T cells. **(A)** NPM1 deficiency promotes the proliferation of BMDMs cultured with MC38 conditioned medium. **(B–D)** Flow cytometry analysis of the percentage of CD4^+^ T cells and CD8^+^ T cells in MC38 tumors, representative FACS plots (*n* = 5per group). **(B)** with quantification **(C, D)** were shown (n=5 per group). **(E–J)** Flow cytometry analysis of the GZMB^+^/IFNγ^+^/TNFα^+^ CD8^+^ T cells in MC38 tumors, representative FACS plots **(E–G)** and quantification **(H–J)** were shown. **(L, M)** Flow cytometry analysis of the percentage of CD4^+^ T cells and CD8^+^ T cells in LLC tumors, representative FACS plots **(K)** with quantification **(L, M)** were shown (n=5 per group). **(N–S)** Flow cytometry analysis of the GZMB^+^/IFNγ^+^/TNFα^+^ CD8^+^ T cells in LLC tumors, representative FACS plots **(N–P)** and quantification **(Q–S)** were shown. One-way ANOVA with Tukey’s *post-hoc* test was used to evaluate statistical differences among groups, *p < 0.05, **p < 0.01, ***p < 0.001, ****p < 0.0001.

Supplementary Figure 4 Macrophage depletion abrogates NPM1-mediated regulatory effects on tumor growth. **(A)** Schematic diagram of the MC38 tumor model (*n* = 5per group). **(B)** Representative image of tumor growth in *Npm1*^+/+^ and *Npm1*^+/-^mice with or without CL subcutaneously inoculated with MC38 cells (*n*= 5 per group). **(C)** Comparison of MC38 tumor weight from two groups at the end of the experiment. One-way ANOVA with Tukey’s *post-hoc* test was used to evaluate statistical differences among groups, *p < 0.05, **p < 0.01, ***p < 0.001, ****p < 0.0001.

Supplementary Figure 5 The effects of NPM1 expression on CCL2 and macrophage polarization markers. **(A, B)** The expression of CCRL2 in BMDMs from *Npm1*^+/-^ and *Npm1^UTR-/^*^-^mice. **(C)** qPCR assays were used to analyze the expression of M1-type or M2-type macrophage markers in BMDMs treated with tumor-conditioned medium. Statistical differences were analyzed by unpaired t-test. *p < 0.05, **p < 0.01.

Supplementary Figure 6 Overexpression of *Npm1* inhibite the infiltration and function of CD8^+^ T cells. **(A–C)** Flow cytometry analysis of the percentage of CD4^+^ T cells and CD8^+^ T cells in MC38 tumors, representative FACS plots (*n* = 5per group). **(A)** with quantification **(B, C)** were shown (n=6 per group). **(D–I)** Flow cytometry analysis of the GZMB^+^/IFNγ^+^/TNFα^+^ CD8^+^ T cells in MC38 tumors, representative FACS plots **(D–F)** and quantification **(G–I)** were shown. **(J–L)** Flow cytometry analysis of the percentage of CD4^+^ T cells and CD8^+^ T cells in LLC tumors, representative FACS plots **(J)** with quantification **(K, L)** were shown (n=5 per group). **(M–R)** Flow cytometry analysis of the GZMB^+^/IFNγ^+^/TNFα^+^ CD8^+^ T cells in LLC tumors, representative FACS plots **(M–O)** and quantification **(P–R)** were shown. Statistical differences were analyzed by unpaired t-test. **p < 0.01, ***p < 0.001.
