# Supplementary material for: Macrophage NPM1 drives anti-tumor immunity via the STAT3-CCRL2 axis
Source: Front Immunol. 2026 Jul 22;17:1748791. doi: 10.3389/fimmu.2026.1748791 (PMC13439533; doi:10.3389/fimmu.2026.1748791)
Supplement: Supplementary file 1 [file Table1.docx]

**Supplementary Table 1 Primer Sequences**

| Gene name | Forward primer (5'→3') | Reverse primer (5'→3') |
| --- | --- | --- |
| m-CD86 | ACGTATTGGAAGGAGATTACAGCT | TCTGTCAGCGTTACTATCCCGC |
| m-iNOS | GAGACAGGGAAGTCTGAAGCAC | CCAGCAGTAGTTGCTCCTCTTC |
| m-TNFα | GGTGCCTATGTCTCAGCCTCTT | GCCATAGAACTGATGAGAGGGAG |
| m-IL1B | TGGACCTTCCAGGATGAGGACA | GTTCATCTCGGAGCCTGTAGTG |
| m-NPM1 | GCACATTAGTGGACAGCATCTAG | GTTACCACCTCCAGGAGCAGAT |
| m-ARG1 | CATTGGCTTGCGAGACGTAGAC | GCTGAAGGTCTCTTCCATCACC |
| m-CD206 | GTTCACCTGGAGTGATGGTTCTC | AGGACATGCCAGGGTCACCTTT |
| m-IL10 | CGGGAAGACAATAACTGCACCC | CGGTTAGCAGTATGTTGTCCAGC |
| H-CD86 | CCATCAGCTTGTCTGTTTCATTCC, | GCTGTAATCCAAGGAATGTGGTC |
| H-iNOS | GCTCTACACCTCCAATGTGACC | CTGCCGAGATTTGAGCCTCATG |
| H-TNFα | CTCTTCTGCCTGCTGCACTTTG, | CCACAGACCTTCCAGGAGAATG |
| H-IL1B | CCACAGACCTTCCAGGAGAATG | GTGCAGTTCAGTGATCGTACAGG; |
| H-ARG1 | TCATCTGGGTGGATGCTCACAC | GAGAATCCTGGCACATCGGGAA |
| H-CD206 | AGCCAACACCAGCTCCTCAAGA | CAAAACGCTCGCGCATTGTCCA |
| H-IL10 | TCTCCGAGATGCCTTCAGCAGA | TCAGACAAGGCTTGGCAACCCA |
